# Acceptability of HIV self-testing is low among men who have sex with men who have not tested for HIV: a study with respondent-driven sampling in Brazil

**DOI:** 10.1186/s12879-020-05589-0

**Published:** 2020-11-19

**Authors:** Laio Magno, Andrea Fachel Leal, Daniela Knauth, Inês Dourado, Mark Drew Crosland Guimarães, Elis Passos Santana, Tiago Jordão, Gustavo Machado Rocha, Maria Amélia Veras, Carl Kendall, Alexandre Kerr Pontes, Ana Maria de Brito, Ligia Kerr, The Brazilian HIV/MSM Surveillance Group, Alexandre K. Pontes, Ana C. Camillo, Ana M. Brito, Ageu Magalhães, Ana R. C. Motta-Castro, Daniela R. Knauthe Andréa F Leal, Edgar Merchan-Hermann, Ximena P. Diaz, Luana N. C. Lima, Maria A. Veras, Inês Dourado, Lígia R. F. S. Kerr, Lisangela C. Oliveira, Mark D. C. Guimarães, Raimunda H. M. Macena, Rosa S. Mota, Maria S. Cavalcante, Carl Kendall, George Rutherford, Willi McFarland

**Affiliations:** 1 Department of Life Sciences, Bahia State University, Rua Silveira Martins, 2555, Cabula, Salvador, 41.150-000 Bahia Brazil; 2 Institute of Collective Health, Federal University of Bahia, R. Basílio da Gama, s/n - Canela, Salvador, 45760-030 Bahia Brazil; 3 Sociology Department, Universidade Federal do Rio Grande do Sul, Porto Alegre, Brazil; 4 Department of Social Medicine, Universidade Federal do Rio Grande do Sul, School of Medicine, Porto Alegre, Brazil; 5 Department of Preventive and Social Medicine, Federal University of Minas Gerais, Belo Horizonte, Brazil; 6 Universidade Federal de São João Del-Rei, Divinópolis, Brazil; 7 Santa Casa de São Paulo School of Medical Sciences, São Paulo, Brazil; 8 Tulane School of Public Health and Tropical Medicine, New Orleans, USA; 9 Department of Community Health, Federal University of Ceará, Fortaleza, Brazil; 10 Federal University of Rio de Janeiro, Rio de Janeiro, Brazil; 11 Aggeu Magalhães Institute, Oswaldo Cruz Foundation, Recife, Brazil

**Keywords:** HIV self-test, Acceptability, HIV testing, Men who have sex with men, Respondent-driven sampling, Brazil

## Abstract

**Background:**

Brazil has many people living with HIV (PLWH) who are unaware of their serostatus. The public health system has recently added HIV self-testing (HIVST) for key populations such as men who have sex with men (MSM). This study estimates HIVST acceptability among Brazilian MSM and explores factors associated with acceptability among MSM who have never tested for HIV or who had a previous negative result.

**Methods:**

Respondent-driven sampling (RDS) was used to recruit 4176 MSM in 12 Brazilian cities in 2016 to this biological and behavioral surveillance study. We excluded from this analysis all MSM who were aware of their positive HIV serostatus. Descriptive, bivariate and multivariate analyses were conducted. Overall proportions were weighted with Gile’s estimator in RDS Analyst software and 95% confidence intervals were calculated. The analyses of HIVST acceptability were stratified by prior HIV testing (never or one or more times).

**Results:**

For this analysis, 3605 MSM were included. The acceptability of HIVST was 49.1%, lower among those who had never tested for HIV (42.7%) compared to those who had a previous HIV negative test (50.1%). In the subgroup of MSM who had never tested for HIV, those who reported discrimination or who had a medical appointment in the last 12 months reported higher HIVST acceptability. Among MSM who had a previous negative HIV test, only those reporting condomless receptive anal sex reported higher HIVST acceptability. In addition, we observed that high levels of knowledge of HIV/AIDS, taking part in lesbian, gay, bisexual, and transgender nongovernmental organizations (LGBT-NGO), or complete secondary or incomplete higher undergraduate education reported higher acceptability.

**Conclusions:**

The acceptability of HIVST was low among MSM, especially among those who never tested for HIV. Given access to HIVST in Brazil, we point to the need for programs that enhance promotion of testing addressed to MSM.

## Background

Routine testing to detect HIV infection is an important measure for a combined HIV prevention strategy [1] since it encompasses three levels of intervention. First, a biomedical level, since technology is used to detect the virus and initiate antiretroviral therapy (ART), interrupting transmission [2]. Second, a behavioral and cultural level, embedding individual and group decisions to test in a psycho-social matrix of sex, risk and prevention [3]. Finally, successful testing depends not only on the availability of the test, but also on overcoming barriers such as stigma and discrimination when accessing testing services [4, 5].

Lack of awareness of HIV status affects access to ART and consequently produces higher viral loads, a potential factor for HIV transmission [6]. In many countries, men who have sex with men (MSM) are disproportionately affected by HIV/AIDS compared to the general population [7].

In Latin America, there are 1.9 million PLWH and 80% of these knew their status in 2018 [8]. It has been estimated that in Brazil there are 900,000 PLWH and that approximately 135,000 PLWH are unaware of their serostatus. This corresponds to 85% of the 90% goal of the 90–90-90 UNAIDS goals for PLWH [9, 10].

A survey using respondent-driven sampling (RDS) among MSM in 12 state capitals in 2016 indicated that Brazilian MSM knowledge of HIV serostatus is low. Among those who were tested and diagnosticated living with HIV in this study, 44% had not been aware of their previous serostatus [11]. A similar RDS study, conducted in 2009 in 10 cities around the country demonstrated that almost half (48%) of MSM had never previously tested for HIV [12]. In a comparison between the 2009 and 2016 surveys, there was a drop in the proportion of MSM who had never tested, from 49.8% in 2009 to 33.8% in 2016, and an increase in the proportion of those who tested during the previous 12 months, from 21.2 to 43.3% [13]. In Salvador, a capital city in the country’s most impoverished region (Northeast), in the 2009 survey, 62.8% of MSM reported they had never tested for HIV [14]. In Curitiba, a city in a high-income region (South), in another survey among MSM in 2015, the proportion never tested was much smaller: 24.3% [15], demonstrating important geographical disparities in rates of HIV testing among MSM in Brazil.

In Brazil, free access to HIV test occurs mainly in specialized services (e.g. testing and counseling centers, clinics and hospitals). Recently, HIV testing has been made available in Primary Health Care (PHC) clinics in the Brazilian National Health System (*Sistema Único de Saúde: SUS*) [16]. However, the primary beneficiaries of this program are pregnant women [17], and coverage among sexual minorities remains low [18, 19]. Stigma and discrimination towards MSM in Brazil are high, and is considered a barrier to HIV testing [20–22]. One option to respond to this deficit is HIV self-testing (HIVST). HIVST has been introduced in many countries, including Brazil, following a recommendation from the World Health Organization in 2016 [23]. HIVST is simple, easy to use, allows the individual to conduct the test alone, can use oral fluid or a blood sample, and results can be interpreted without professional help [23].

HIVST guarantees confidentiality and privacy [24, 25]. By enabling testing outside the health services HIVST decentralizes testing and strengthens people’s autonomy [23, 26]. Furthermore, it may facilitate an increase in testing frequency among individuals at high risk of HIV infection and in populations groups with less frequency of testing in traditional services [24]. A meta-analysis showed that HIVST doubled uptake of testing among men, frequency of testing among MSM, and the likelihood of an HIV-positive diagnosis [27]. HIVST could also improve HIV care as it allows for early detection and treatment initiation [28] and can potentially reduce the spread of HIV infection through test and treat [2, 29, 30], and increased HIV risk perception [31].

There are few studies on the acceptability of HIVST in middle-income countries [25]. In Latin America, an RDS survey in Argentina showed that 74% of the MSM were likely to buy an HIVST at a pharmacy for use at home [32] and a cross-sectional online survey in Brazil among MSM previously testing HIV negative estimated that 47% reported a preference for an HIVST at home [33]. More recently, two cross-sectional online surveys, carried out on a sample of MSM users of hookup apps from five Brazilian regions, estimated that HIVST awareness ranged from 26% in 2016 to 33% in 2017, and willingness to use from 50 to 42%, respectively [34]. Although these studies have assessed HIVST acceptability among MSM, few have explored how previous testing affects acceptability, especially when comparing those who never tested to those who had a previous negative HIV result. Both groups could potentially benefit from access to additional testing services [35]. Moreover, there are no studies of HIVST acceptability among a large population sample of Brazilian MSM. Therefore, this article aims to estimate the acceptability of HIVST among MSM from 12 Brazilian cities and to investigate factors associated with acceptability among those never tested for HIV or who had a previous HIV negative result.

## Methods

### Study location, design, and population

The study is based on data collected in a behavioral and biological surveillance study entitled “A nationwide study of the behaviors, attitudes, practices, and prevalence of HIV, Syphilis and Hepatitis B and C among MSM in Brazil”, conducted from June to December 2016 in 12 state capitals: Manaus and Belém in the Northern region; Fortaleza, Recife and Salvador, in the Northeast; Brasília and Campo Grande in the Central-West; Belo Horizonte, Rio de Janeiro and São Paulo in the Southeast; and Curitiba and Porto Alegre in the South.

The sample consisted of men defined as biologically male, 18 years of age or older, reporting oral sex or anal intercourse with another man in the previous 12 months; and living, studying or working in one of the study cities.

### Data collection

RDS methods were used to recruit participants. Before initiation of the survey, formative qualitative research using both individual interviews and focus group discussions was conducted to explore participation in the study, including willingness to recruit, testing, study logistics, such as level and kind of incentive, siting, and working hours [36].

To start the recruitment process six MSM were purposively selected in each city. These individuals, known as seeds, had large social networks of MSM and represented different sociodemographic characteristics. Next, each of these seeds invited three other MSM from their social networks using three invitation coupons. This procedure was repeated in sequence until the desired sample size was reached, estimated a priori as 350 participants per city. Participants received BRL 25.00 (USD $7.40) for their participation and for each recruited participant (BRL 25.00 for each one up to a total of three new participants - BRL 100.00) as a means of reimbursing their expenses for transportation and food. Data were collected using computer assisted personal interviews (CAPI) at fixed office sites, where educational materials, condoms and lube were provided, and where counseling, blood draws and results were provided. A total of 4176 MSM were recruited in the 12 cities, who signed separate consent forms for the interview and for testing. The Research Ethics Committee of the Federal University of Ceará approved the research project. Further details can be found in Kendall et al. [36].

### Study variables

A descriptive analysis was performed for the following variables: (1) demographic and education: age (< 25 years and ≥ 25 years), self-declared race (white, black, brown/mixed race, indigenous/native, Asian origin), schooling (primary and incomplete secondary education, complete secondary education, incomplete undergraduate education, and complete undergraduate education) and marital status (single, separated or widowed; married or living together); (2) socio-economic status with three categories: A-B for high; C for middle; D-E for low) based on the Brazilian Economic Classification Criteria [37]; (3) discrimination: self-reported discrimination due to sexual orientation during lifetime (yes, no); (4) health services: medical appointment in the 12 months before the study (yes, no), having taken part in any workshop about sexually transmitted infections (STI) and HIV/AIDS in the 12 months before the study (yes, no); and ever testing for HIV was determined by responses to the following question: “have you ever been tested for HIV/AIDS in your life?” (yes, no); (5) knowledge about HIV/AIDS (low, middle and high) was analyzed according to item response theory (IRT), measured using 12 items about transmission and prevention (i.e. there are medicines for HIV-negative people to take to prevent HIV; an HIV-infected person who is taking AIDS medication has a lower risk of transmitting the virus, etc.), as described in Guimarães et al. [38]; (6) self-perception of risk (low, middle and high), in response to the question: “how do you assess your chance of becoming infected with HIV during your lifetime?”; (7) participation in an LGBT nongovernmental organization (NGO) (yes, no); and (8) condomless receptive anal sex in past 6 months (always used condoms and irregular use of condoms).

The outcome HIVST acceptability (yes, no) was structured according to the following question: “would you use an HIV/AIDS diagnosis test that you applied to yourself?”. The reasons for the decision to use or not use an HIVST were also collected.

### Data analysis

We excluded MSM who were aware of their positive HIV serostatus before the study, yielding 3605 MSM for this analysis. The dependence between observations resulting from recruitment chains in RDS, i.e., the probability of unequal selection and the different sizes of each participant’s contact network [39], was taken into account. Gile’s estimator [40] was used to weigh the proportion estimates with a 95% confidence interval using RDS Analyst [41]. We aggregated all data from the 12 cities into a single dataset with each city serving as a stratum.

The weighted data were analyzed using the complex sample routines in STATA 15 (StataCorp, College Station, TX, USA). Descriptive, bivariate analyses were conducted using frequency distributions of the variables of interest and the differences between the analyzed proportions using Pearson’s χ^2^ test. All analyses were stratified according to prior HIV testing. i.e., those who were never tested or those who were tested at least once in a lifetime and their HIV result was negative. A multivariate logistic regression model was constructed to estimate the Odds Ratio (OR) with the associated 95% confidence intervals (CI), as a measure of association between the potential associated factors and HIVST acceptability. Selection of variables for multivariate modeling was based on the bivariate analysis (*p*-value < 0.15) and the epidemiological relevance, taking into account a previous literature review, concerning potential factors associated with HIVST acceptability. Those variables with a *p*-value of < 0.05 remained in the final model.

## Results

Table 1 shows descriptive results by 1) no previous HIV test and 2) a previous negative HIV result. The following characteristics did not differ significantly by the two categories: MSM under 25 years of age (64.3 and 59.5%, respectively), brown/mixed race (48.4 and 39.2%, respectively), single, separated/widowed (90.1 and 86.1%, respectively), and reporting protective sex (66.8 and 61.3%, respectively) (*p* > 0.05). MSM with no previous HIV test, compared to MSM who had a previous negative HIV result, had less schooling (46.1% vs 23.5%, *p* < 0.001), lower socio-economic status (26.5% vs 9.7%, p < 0.001), reported less experience of discrimination during lifetime (53.1% vs 71.5%, *p* < 0.001) and had lower HIV risk perception (24.9% vs 14.6%, *p* < 0.01). In terms of health services, the majority of MSM did not engage in workshops on STI and HIV/AIDS in the last 12 months in both groups (83.0 and 79.0%, respectively, *p* > 0.05). On the other hand, those who never tested reported fewer medical appointments in the last 12 months (69.6% vs 87.1%, *p* < 0.001), lower knowledge about HIV/AIDS (35.4% vs 24.0%, p < 0.001) and participated less in LGBT NGO (12.1% vs 19.9%, *p* = 0.03) than those who had tested negative.

**Table 1:** Descriptive analysis of MSM according to no previous HIV test and a previous negative HIV result Brazil, 2016

Variables	No previous HIV test^a^			Previous negative HIV result^a^			*p*-value
	%	95%CI		%	95%CI		
**Age**							0.26
< 25 years old	64.3	(57.2–70.9)		59.5	(54.8–64.1)		
≥ 25 years old	35.7	(29.1–42.8)		40.5	(35.9–45.2)		
**Race**							0.09
Brown/mixed race	48.4	(41.6–55.2)		39.2	(34.7–43.9)		
White	29.1	(22.9–36.2)		34.9	(30.5–39.5)		
Black	22.5	(17.9–27.9)		25.9	(21.4–31.2)		
**Schooling**							< 0.001
Primary/Incomplete secondary education	46.1	(39.6–52.6)		23.5	(19.9–27.5)		
Complete secondary/Incomplete undergraduate education	48.8	(42.4–55.2)		62.6	(58.1–67.0)		
Complete undergraduate education	5.2	(2.3–11.5)		13.9	(11.3–17.1)		
**Socio-economic status groups**							< 0.001
D-E (Low)	26.5	(21.0–32.8)		9.7	(7.5–12.4)		
C (Middle)	38.5	(32.3–45.0)		42.1	(37.4–46.9)		
A-B (High)	35.1	(29.1–41.6)		48.2	(43.5–53.0)		
**Marital status**							0.10
Single/separated/widowed	90.1	(85.9–93.2)		86.1	(82.9–88.8)		
Married/living together	9.9	(6.8–14.1)		13.9	(11.2–17.1)		
**Self-reported discrimination during the lifetime**							< 0.001
No	46.9	(40.5–53.5)		28.5	(24.7–32.7)		
Yes	53.1	(46.5–59.5)		71.5	(67.3–75.3)		
**Medical appointment in the last 12 months**							< 0.001
No	30.4	(25.0–36.4)		12.9	(10.2–16.1)		
Yes	69.6	(63.6–75.0)		87.1	(83.8–89.7)		
**Took part of any workshop on STI and HIV/AIDS in the last 12 months**							0.26
No	83.0	(76.7–87.9)		79.0	(74.8–82.7)		
Yes	17.0	(12.1–23.3)		21.0	(17.3–25.2)		
**Knowledge about HIV/AIDS (IRT score)**^**2**^							< 0.001
Low	35.4	(29.4–41.9)		24.0	(19.6–29.1)		
Middle	47.6	(41.0–54.2)		47.0	(42.3–51.8)		
High	17.1	(12.7–22.6)		29.0	(25.0–33.3)		
**Self-perception of risk**							< 0.01
Low	24.9	(19.6–31.0)		14.6	(11.6–18.4)		
Middle	60.8	(54.1–67.8)		74.8	(70.2–79.0)		
High	14.2	(9.4–20.9)		10.5	(7.7–14.1)		
**Took part of LGBT NGO**							0.03
No	87.9	(81.7–92.2)		80.1	(76.2–83.6)		
Yes	12.1	(7.8–18.3)		19.9	(16.5–23.8)		
**Condomless receptive anal sex (past 6 months)**							0.15
Always used condoms	66.8	(60.5–72.4)		61.3	(56.8–65.6)		
Irregular use of condoms	33.3	(27.6–39.5)		38.7	(34.4–43.2)		

^a^ Weighted by Gile-SS

Overall HIVST acceptability was 47.3% (95% CI: 43.5, 51.1) with reasons given: curiosity (31.3%), as a routine test (27.8%), easiness (17.8%), they were at risk for HIV infection (16.6%), and confidentiality (11.3%). And reasons for not using it were: fear (42.7%), did not see why use it (23.8%), unaware of test availability (2.3%), believed to be at low or no risk for HIV infection (1.8%) and because it has never been offered (1.6%) (Table 2).

**Table 2:** Acceptability of HIVST and reasons for use/non-use among MSM according to no previous HIV test and a previous HIV negative result in 12 Brazilian cities, 2016

Variables	Overall^a^			No previous HIV test^a^			Previous negative HIV result^a^			*p*-value*
	%	95%CI		%^2^	95%CI		%	95%CI		
**Acceptability of HIVST**										0.07
Yes	47.3	(43.5–51.1)		42.7	(36.3–49.3)		50.1	(45.3–54.9)		
No	52.7	(48.9–56.5)		57.3	(50.7–63.7)		49.9	(45.1–54.7)		
**Reasons to use HIVST**										
Curiosity	31.3	(26.7–36.4)		36.8	(27.1–47.7)		28.6	(24.0–33.7)		0.14
As a routine test	27.8	(24.2–31.7)		25.8	(19.2–33.8)		28.8	(24.7–33.4)		0.50
It is more practical/easiness	17.8	(13.6–22.8)		10.7	(6.3–17.5)		21.4	(15.9–28.1)		0.01
Believes to be at risk for HIV infection	16.6	(13.5–20.4)		16.8	(11.0–24.8)		16.6	(13.1–20.8)		0.95
Confidentiality	11.3	(8.5–14.9)		8.9	(6.1–12.8)		12.6	(8.8–17.6)		0.18
**Reasons to not use HIVST**										
Fear	42.7	(37.2–48.3)		50.0	(41.8–58.3)		37.8	(30.8–45.3)		0.03
Does not see any reason to use it	23.8	(20.0–27.9)		24.6	(19.0–31.2)		23.2	(18.3–28.9)		0.73
Unaware of test availability	2.3	(1.6–3.3)		2.5	(1.5–4.4)		2.1	(1.2–3.5)		0.62
Believe to be at low or no risk for HIV infection	1.8	(1.0–3.0)		1.1	(0.5–2.3)		2.2	(1.1–4.4)		0.14
Because it has never been offered	1.6	(1.0–2.6)		1.2	(0.6–2.4)		1.8	(1.0–3.4)		0.39

^a^ Weighted proportion by Gile-SS

* *p*-value refers to the comparison of proportions between: “no previous HIV test” and “negative previous HIV result”

HIVST acceptability was higher among those who had a previous negative HIV result (50.1%; 95% CI: 45.3, 54.9), as compared to those with no previous HIV test (42.7%; 95% CI: 36.3, 49.3), but this did not reach statistically significance (*p* = 0.07). Regarding reasons for its use and non-use, respectively, only easiness of the test (10.7% vs 21.4%, *p* = 0.01) and fear (50.0% vs 37.8%, *p* = 0.03) were significantly different when comparing the two groups (Table 2).

Table 3 shows the bivariate and multivariate analyses also stratified by testing history. In the multivariate analysis, among MSM with no previous HIV test, experience of discrimination (AOR = 2.00, 95% CI: 1.18, 3.38) and a medical appointment in the last 12 months (AOR = 1.74, 95% CI: 1.01, 2.99) had a higher odds of HIVST acceptability. Among MSM who had a previous HIV negative test, condomless receptive anal sex (AOR = 1.46, 95% CI: 1.00, 2.15) had greater odds of HIVST acceptability. In addition, in both strata, we observed that high levels of HIV/AIDS knowledge (AOR = 2.45, 95% CI: 1.05, 5.70 and AOR = 2.79, 95% CI: 1.58, 4.93), participating in an LGBT NGO (AOR = 3.26, 95% CI: 1.29, 8.25 and AOR = 2.06, 95% CI: 1.28, 3.32), and complete secondary or incomplete undergraduate education (AOR = 1.74, 95% CI: 1.01, 3.01 and AOR = 2.17, 95% CI: 1.34, 3.51) were associated with greater odds of HIVST acceptability.

**Table 3:** Acceptability of HIVST among an RDS sample of MSM. stratified by the report of prior HIV testing in 12 Brazilian cities. 2016

Variables	No previous HIV test					Previous negative HIV result				
	Bivariate			Multivariate		Bivariate			Multivariate	
	Wt%^a^	Unadj OR^b^	*p*-value	AdjOR^c^	*p*-value	Wt%^a^	Unadj OR^b^	*p*-value	AdjOR^c^	*p*-value
**Age**										
≥ 25 years	34.2	1.00	0.07			48.3	1.00	0.53		
< 25 years	48.3	1.79 (0.93–3.45)		–	–	51.4	1.13 (0.76–1.66)		–	–
**Race**										
Black	34.6	1.00	0.30			49.9	1.00	0.09		
Brown/mixed race	44.5	1.51 (0.83–2.74)		–	–	44.0	0.78 (0.45–1.36)		–	–
White	48.6	1.79 (0.86–3.70)		–	–	57.4	1.35 (0.76–2.40)		–	–
**Schooling**			0.01					< 0.001		
Primary/Incomplete secondary education	35.5	1.00		1.00		30.1	1.00		1.00	
Complete secondary/Incomplete undergraduate education	53.1	2.05 (1.16–3.64)		1.74 (1.01–3.01)	0.04	53.7	2.69 (1.70–4.26)		2.17 (1.34–3.51)	< 0.001
Complete undergraduate education	20.4	0.46 (0.11–1.93)		0.86 (0.27–2.76)	0.81	67.4	4.80 (2.58–8.92)		3.39 (1.75–6.56)	< 0.001
**Socio-economic status groups**										
D-E (Low)	32.5	1.00	0.07			27.0	1.00	< 0.001		
C (Middle)	40.6	1.41 (0.67–2.96)		–	–	48.5	2.54 (1.47–4.36)		–	–
A-B (High)	52.6	2.30 (1.08–4.89)		–	–	56.1	3.45 (2.02–5.88)		–	–
**Marital status**										
Married/living together	43.7	1.00	0.91	–	–	55.2	1.00	0.33	–	–
Single/separated/widowed	42.6	0.95 (0.42–2.16)		–	–	49.2	0.78 (0.47–1.28)		–	–
**Self-report of discrimination during the lifetime**										
No	29.3	1.00	< 0.001	1.00		38.8	1.00	0.001	–	–
Yes	54.4	2.87 (1.69–4.87)		2.00 (1.18–3.38)	0.01	54.6	1.89 (1.27–2.82)		–	–
**Medical appointment in the last 12 months**										
No	33.6	1.00	0.01	1.00		48.6	1.00	0.75		
Yes	49.9	1.96 (1.12–3.40)		1.74 (1.01–2.99)	0.04	50.7	1.08 (0.64–1.82)		–	–
**Took part of any workshop on STI and HIV/AIDS in the last 12 months**										
No	39.1	1.00	0.03			53.1	1.00	0.03		
Yes	58.3	2.17 (1.03–4.57)		–	–	41.2	0.61 (0.39–0.96)		–	–
**Knowledge about HIV/AIDS (IRT score)**^**d**^										
Low	38.3	1.00	0.06	1.00		31.7	1.00	< 0.001	1	
Middle	39.8	1.06 (0.57–1.98)		1.13 (0.66–1.96)	0.64	52.6	2.39 (1.37–4.14)		2.38 (1.44–3.92)	< 0.001
High	59.9	2.41 (1.08–5.37)		2.45 (1.05–5.70)	0.04	61.2	3.39 (1.85–6.24)		2.79 (1.58–4.93)	< 0.001
**Self-perception of risk**										
Low	38.0	1.00	0.67			37.3	1.00	0.11		
Middle	45.9	1.38 (0.72–2.62)		–	–	51.7	1.79 (0.95–3.37)		–	–
High	42.6	1.21 (0.40–3.64)		–	–	56.4	2.17 (0.91–5.15)		–	–
**Took part of LGBT NGO**										
No	39.5	1.00	0.02	1.00		46.6	1.00	< 0.001	1.00	
Yes	65.0	2.84 (1.12–7.17)		3.26 (1.29–8.25)	0.01	64.1	2.04 (1.23–3.39)		2.06 (1.28–3.32)	0.003
**Condomless receptive anal sex (past 6 months)**										
Always used condoms	42.5	1.00	0.96			45.1	1.00	0.01	1.00	
Irregular use of condoms	42.8	1.01 (0.57–1.76)		–	–	56.5	1.58 (1.09–2.28)		1.46 (1.00–2.15)	0.049

^a^ Gile-SS weighted proportion of acceptability

^b^ Unadjusted weighted odds ratio of acceptability with 95% confidence limits

^c^ Adjusted weighted odds ratio of acceptability with 95% confidence limits

^d^ Score obtained through Item Response Theory Analysis

## Discussion

The overall acceptability of HIVST was close to 50%, but it was lower among those with no previous HIV test and higher among MSM who were better off economically and better educated (i.e. those with higher level of schooling and socio-economic status, higher HIV/AIDS knowledge and who had a medical appointment in the last 12 months). This may argue that promotion of HIVST in Brazil should be more focused on MSM at higher social vulnerability, that is, MSM with lower levels of schooling, less HIV/AIDS knowledge and poorer access to health services.

Figueroa et al. [25], in a systematic review of HIVST acceptability among key-populations, demonstrated that, of the 14 studies analyzed, eight indicated high (≥67%), five moderate (between 34 and 66%), and one low (≤33%) acceptability of HIVST. Moderate acceptability found in our study may be due to the date of our field work in 2016, before HIVST was widely available in Brazil, either commercially or through the Brazilian National Health System (*SUS*). While the National Health Surveillance Agency (*Agência Nacional de Vigilância Sanitária*: ANVISA) approved the sale of HIVST in pharmacies in 2015, the first HIVST product was registered in 2017. The product has been commercially available since then, in both physical and online drugstores in all Brazilian states, at a cost of BRL 70.00 (21.90 US$) or BRL 80.00 (25.00 US$) per kit (7 to 8% of the monthly minimum wage). It is noteworthy that even among those willing to use an HIVST, its high cost by Brazilian standards in the private sector may act as a barrier and compromise the potential role of this strategy, particularly in low-income settings, thereby justifying free distribution to key and priority populations in the public sector [42].

Brazil until recently, had a long track record of successfully implementing HIV prevention and treatment interventions through the Brazilian National Health System (*SUS*) [43–45]. In 2019, Brazil began offering HIVST without cost in 14 selected cities and is now expanding to other cities. The strategy initially adopted was the distribution of HIVST in familiar venues for key-populations and those highly vulnerable to HIV infection [46]. The implementation of free distribution of HIVST may promote an increase of testing frequency among high-risk MSM [47]. Moreover, it has been identified as a cost-effective prevention technology [48, 49].

HIVST has been advanced as a strategy to enhance testing and contribute to the reduction of HIV transmission [26, 27, 50]. Our results suggest that the acceptability of HIVST is higher precisely among those who have already tested for HIV, who therefore already have access to some form of health service. We believe public policy for HIV combined prevention should focus on expanding HIVST to MSM with less access to traditional health services.

We also found greater acceptability among those who knew more about HIV/AIDS, took part in an LGBT NGO and with a recent medical appointment. This highlights the role of both demand creation campaigns directed to MSM and support for MSM-friendly NGO, which unfortunately have been under- and defunded in the past several years in Brazil [51, 52]. HIVST should be made available with reinforced HIV/AIDS prevention activities that promote HIVST within a combined prevention strategy, including strengthening and extending health services, targeted health education campaigns and support for community-based organizations and nongovernmental organizations (NGO) [53].

Of the MSM we interviewed who had no previous HIV test, self-reported discrimination was one of the factors that increased the likelihood of HIVST acceptability. As pointed out in the literature, stigma, concerns about confidentiality and privacy are among the barriers to testing confronted by MSM [4, 53–55]. Those who have not yet been tested, or were tested a long time ago, may resort to HIVST as a strategy to avoid potential discrimination or confidentiality fears in health services. On the other hand, among MSM who had a previous HIV negative test, those who reported condomless receptive anal sex were more likely to accept an HIVST. A study conducted in Spain showed that MSM reporting high-risk behaviors had greater intentions to test [56].

“Fear” was reported as the main reason for not using HIVST. However, “fear” is not limited to HIVST technology, given that fear is also a barrier to conventional testing conducted in health services [57]. The fear of testing is well documented in many population groups, and amplified here because of the stigma associated with homosexuality [4, 58, 59]. Furthermore, although our data do not allow us to explore fear of using HIVST in-depth, we could argue that it is related to factors found in other studies. Specifically, in the case of HIVST, the literature refers to the fear of a positive result without being properly linked to a health service [54, 58, 60]. As Flowers et al. suggest, in research conducted with MSM in the United Kingdom in 2014, HIVST has the potential to reduce certain barriers related to traditional testing but may create other complicating factors, such as a reduction in linkage to health services and health professionals to follow up test results and care services, and fewer opportunities for prevention and counseling of risk behaviors [55].

Our analysis is not without limitations, including selection bias in the networks recruited and interpretation of results when using RDS. The survey questionnaire did not allow for more specific questions on HIVST such as awareness. Moreover, as a cross-sectional study, full temporal inference is not possible, although some variables are partially supported when referring to events that have occurred in the past.

## Conclusion

Our data suggest greater acceptability of HIVST precisely within the group of MSM who had a previous HIV negative test and were in a less vulnerable social context. Given the acceptability of HIVST and its current policy in Brazil, we suggest improvements that build on our results and expand this strategy especially to more vulnerable MSM, as well as the development of a monitoring system capable of incorporating the results of HIVST and linking MSM to healthcare services. In future studies, it is worth examining health care providers knowledge and acceptance of HIVST, and the dynamics of recruitment and retention of PLWH into services for follow-up. The growing availability of HIVST in Brazilian pharmacies and services of the national health system may affect an already challenged access-to-treatment system. Future studies should also explore price-sensitivity since currently HIVST available in the commercial sector is too expensive for repeated use (i.e. after each risky sexual episode) for many MSM.

## Data Availability

The data that support the findings of this study are available in: https://dataverse.harvard.edu/dataset.xhtml?persistentId=doi:10.7910/DVN/MCMU3110.7910/DVN/MCMU31

## Abbreviations

PLWH: People living with HIV
HIVST: HIV self-testing
MSM: Men who have sex with men
RDS: Respondent-driven sampling
LGBT-NGO: Lesbian, gay, bisexual, and transgender nongovernmental organizations
ART: Antiretroviral therapy
UNAIDS: Joint United Nations Programme on HIV/AIDS
PHC: Primary Health Care
SUS: Brazilian National Health System
CAPI: Computer assisted personal interviews
CI: Confidence intervals
AOR: Adjusted odds ratio
ANVISA: National Health Surveillance Agency
NGO: Nongovernmental organizations
STI: Sexually transmitted infections
